# The prognostic significance of wild-type isocitrate dehydrogenase 2 (IDH2) in breast cancer

**DOI:** 10.1007/s10549-019-05459-7

**Published:** 2019-10-10

**Authors:** Abrar I. Aljohani, Michael S. Toss, Sasagu Kurozumi, Chitra Joseph, Mohammed A. Aleskandarany, Islam M. Miligy, Rokaya El Ansari, Nigel P. Mongan, Ian O. Ellis, Andrew R. Green, Emad A. Rakha

**Affiliations:** 1 Nottingham Breast Cancer Research Centre, Division of Cancer and Stem Cells, School of Medicine, University of Nottingham, Nottingham, UK; 2 Histopathology Department, Faculty of Medicine, Menoufia University, Shibin El Kom, Egypt; 3 Department of Pharmacology, Weill Cornell Medicine, New York, 10065 USA; 4 Faculty of Medicine and Health Sciences, University of Nottingham, Nottingham, LE12 5RD UK; 5 Department of Histopathology, Nottingham University Hospital NHS Trust, City Hospital Campus, Hucknall Road, Nottingham, NG5 1PB UK

**Keywords:** IDH2, Breast cancer, Progression, LVI, Prognosis

## Abstract

**Background:**

Lymphovascular invasion (LVI) is a prerequisite step in breast cancer (BC) metastasis. We have previously identified wild-type isocitrate dehydrogenase 2 (IDH2) as a key putative driver of LVI. Thus, we explored the prognostic significance of IDH2 at transcriptome and protein expression levels in pre-invasive and invasive disease.

**Methods:**

Utlising tissue microarrays from a large well annotated BC cohort including ductal carcinoma in situ and invasive breast cancer (IBC), IDH2 was assessed at the transcriptomic and proteomic level. The associations between clinicopathological factors including LVI status, prognosis and the expression of IDH2 were evaluated.

**Results:**

In pure DCIS and IBC, high IDH2 protein expression was associated with features of aggressiveness including high nuclear grade, larger size, comedo necrosis and hormonal receptor negativity and LVI, higher grade, larger tumour size, high NPI, HER2 positivity, and hormonal receptor negativity, respectively. High expression of IDH2 either in mRNA or in protein levels was associated with poor patient’s outcome in both DCIS and IBC. Multivariate analysis revealed that IDH2 protein expression was an independent risk factor for shorter BC specific-survival.

**Conclusion:**

Further functional studies to decipher the role of IDH2 and its mechanism of action as a driver of BC progression and LVI are warranted.

**Electronic supplementary material:**

The online version of this article (10.1007/s10549-019-05459-7) contains supplementary material, which is available to authorised users.

## Introduction

Breast cancer (BC) progression is a complex multifactorial process. However, the invasion machinery which is a critical step in progression from in situ to infiltrating tumour followed by distant metastasis remains unclear. Deciphering the transcriptomics and proteomics that govern the invasive and metastatic cascades of BC is essential in understanding the mechanisms of cancer progression. Lymphovascular Invasion (LVI) is an independent prognostic factor of poor outcome in invasive BC (IBC) and is a prerequisite for the tumour metastasis [1–4]. Understanding the molecular mechanisms underlying BC progression, and in particular LVI, and unveiling their driver molecular pathways could ultimately improve patient outcomes [5]. Through stringent bioinformatics analysis, we have previously interrogated transcriptomic datasets of IBC [Molecular Taxonomy of Breast Cancer International Consortium (METABRIC) and The Cancer Genome Atlas (TCGA)] for putative drivers of LVI [6]. Briefly, LVI positive and negative cases from these cohorts were subjected to a method of weighted average difference (WAD) and subsequently differentially expressed genes (DEGs) were identified based on WAD rankings [7]. Forty-two significantly overexpressed and 57 downregulated genes identified in the METABRIC cohort were identified and validated in the TCGA cohort [8]. Wild-type Isocitrate Dehydrogenase 2 (*IDH2*) was a highly expressed gene associated with presence of LVI [8]. Moreover, *IDH2* was reported to be differentially expressed between recurrent and non-recurrent DCIS and associated with DCIS recurrence and progression to invasive disease [9, 10].

IDH2 is a member of the isocitrate dehydrogenase family that plays a key role in cellular metabolism and acts in the tricarboxylic acid (TCA) cycle as an NADP + consuming enzyme, producing NADPH. In the reverse TCA cycle, when IDH2 causes reductive carboxylation of 2-oxoglutarate (2-OG), it consumes cell during hypoxia to survive the lower glucose levels [11].

Cellular energy and biosynthetic intermediates are produced by the TCA cycle, which are upregulated in metastasised cancer cells. Glycolysis is also upregulated in cancer cells to produce biosynthetic intermediates and energy needed for cellular proliferation and survival. Circulating tumour cells are predisposed to anoikis as a result of impaired glucose uptake. Thus, the metastasised tumour cells evade anoikis by upregulation of the TCA cycle [12]. Previous studies have reported upregulation of wild-type IDH2 in lung cancer, ovarian cancer, endometrioid carcinoma, and advanced colorectal cancer [13, 14]. Gain of function mutations in IDH2 result in an increase in the oncometabolite 2-hydroxyglutarate (2-HG) which is believed to link aberrant metabolism and aberrant epigenetics and gene regulation in cancer [15]. A well-known function of mutant IDH2 has been demonstrated in cancers such as glioma, cholangiocarcinoma, and breast solid papillary carcinoma with reverse polarity (SPCRP) [16]. Wild-type IDH2 overexpression is an indicator of poor outcome in lung cancer through the stimulation of the Warburg effect to help the maintenance of cancer cells via activation of hypoxia inducible factor 1α (HIF1α) which supports tumour growth in hypoxic environments [17]. However, the role of wild-type IDH2 in BC is still unclear. In this study, we aimed to assess the expression of wild-type IDH2 in BC and evaluate its role in tumour progression, particularly LVI, and patient outcome.

## Materials and methods

### IHD2 protein expression

#### Study cohorts

Large well-characterised BC cohorts consisting of pure ductal carcinoma in situ (DCIS; *n* = 776) and invasive disease (IBC; *n* = 859) from patients presented between 1998 and 2006 at Nottingham City Hospital, Nottingham, United Kingdom (UK) as previously described [18, 19] were utilised in this study. Patients’ demographic data, tumours’ morphological features, treatment data including surgery, hormonal therapy, radiotherapy and chemotherapy were available for both cohorts. Patients receiving neoadjuvant therapy were excluded in this study. Oestrogen Receptor (ER), Progesterone Receptor (PgR), HER2 status and Ki67 data was available [20, 21]. As per previous publications [20, 22, 23], ER and PgR was defined as ≥ 1%. HER2 positivity was defined when ≥ 10% of tumour cells showed strong membranous staining (score + 3), where chromogenic in situ hybridisation technique (CISH) was used to assess the gene amplification status in borderline cases (+2). BC molecular subtypes (for both DCIS and IBC cohorts) including luminal A (ER+/HER2−; Ki67 < 10%), Luminal B (ER+/HER2−; Ki67⩾10%), HER2-positive class (HER2+ regardless of ER status), and TN (ER−, PgR− and HER2 −) were defined based on IHC profile. For further understanding the molecular interactions of IDH2, available data on basal phenotype (CK5, CK14, and EGFR), EMT related markers (E-cadherin, N-cadherin, P-cadherin, TGF beta, and TWIST2), and glutamine metabolism proteins (SLC1A5, SLC3A2, SLC7A5, GLS, ALDH18A1, ALDH4A1, PRODH) were included in this study as per previous publications [24–28].

The patient records were regularly updated including patient’s outcome and follow-up. Local recurrence free interval (LRFI) in DCIS was defined as the time (in months) between 6 months after the first DCIS surgical removal and the occurrence of ipsilateral local recurrence. Patients had close/positive surgical margins or presented with residual tumour tissue and undergoing re-excision surgery within the first 6 months were not considered as recurrence. Patients who developed contralateral breast event after initial diagnosis of DCIS were censored at the time of occurrence of the contralateral disease. For IBC, data on breast cancer-specific survival (BCSS) was defined as the period (in months) extending from the date of primary surgery to the time of death due to breast cancer, and time to distant metastasis (TTDM) was defined as the period (in months) from primary surgery to occurrence of first distant metastasis.

#### Immunohistochemistry (IHC)

Twenty full face BC tissue sections (including DCIS and IBC) based on different tumour grades, LVI status and histological type were stained using IHC to evaluate the pattern of IDH2 protein expression prior to staining of Tissue Microarrays (TMAs). TMAs were previously prepared using a TMA Grand Master® (3D HISTECH®, Budapest, Hungary) [19, 26].

Primary antibody specificity for the mouse monoclonal anti-wild-type IDH2 antibody (ab55271, Abcam, UK) was validated using Western blot. An array of breast cancer cell line lysates was used, which include MCF-10A, MCF7, and MB-MDA-231 (obtained from the American Type Culture Collection, Rockville, MD, USA). IDH2 antibody was used at a dilution of 1:500, which showed a single specific band at the predicted molecular weight of 47 kDa (Supplementary Fig. 1).

Antigen retrieval was performed based on the manufacturer’s recommendations (citrate buffer pH 6.0 at 1000 W for 20 min using microwave). Expression of IDH2 protein was assessed by IHC using the Novocastra Novolink™ Polymer Detection Systems kit (Code: RE7280-K, Leica, Biosystems, UK), where 4 µm sections were incubated for 60 min with mouse monoclonal IDH2 (dilution 1:500). Normal kidney tissue was used as a positive control, while a negative control was carried out by omitting the primary antibody.

#### Scoring of IDH2 expression

Assessment of IDH2 cytoplasmic expression was performed using the semi-quantitative Histochemical score (H-score), where staining intensity was multiplied by the percentage of representative cells in the tissue for each intensity, producing a range of values between 0 and 300 [29]. All non-representative cores (folded tissue during processing and staining or cores with only normal breast tissue or those containing < 15% tumour cells relative to the whole core area) were excluded from scoring. The scoring was performed by AA blinded to patients’ clinicopathological and outcome, with a subset of cores (~ 10%) scored independently by another scorer (MAA) to calculate the inter-observer concordance. The protein expression of IDH2 was dichotomised by cut-off points generated from X-tile bioinformatics software version 3.6.1 (Yale University, USA) based on BCSS in IBC and LRFI in DCIS. An H-score of 70 was the optimal cut-off value of IDH2 protein expression in IBC, while a H-score of 45 was used to dichotomise DCIS cases into high and low expression.

### IDH2 transcriptomic analysis

Two datasets comprising the METABRIC (*n* = 1980) [6] and TCGA breast carcinoma (TCGA BRCA, *n* = 854) [30] were used to evaluate *IDH2* mRNA expression. The median was used as cut-off to categorise mRNA expression levels into high and low subgroups. For further validation of the prognostic significance of *IDH2* expression in BC, the prognostic analytical module within the publicly available online dataset of Breast Cancer Gene Expression Miner v4.0 (http://bcgenex.centregauducheau.fr) was used.

### Statistical analysis

Statistical analysis was performed using SPSS, version 24 (Chicago, IL, USA). The interclass correlation coefficient (ICC) test was used to assess the concordance rate of the IDH2 scoring between both the observers. The association between IDH2 and various clinicopathological parameters in both cohorts was analysed using Chi-square test. Mann–Whitney test was used to compare between IDH2 expression in DCIS and IBC. The Spearman’s rank correlation coefficient was used to assess the correlation between *IDH2* mRNA and protein expression levels in IBC.

Log rank test and Kaplan–Meier curves were used for univariate survival analysis. Analysis with recurrence in DCIS was carried out for the breast conservative surgery (BCS) treated group (and not for those treated by mastectomy). Cox regression model was used for multivariate analysis. For all tests, a two-tailed *p* value < 0.05 was considered as statistically significant.

## Results

### Patterns of IDH2 protein expression

**Fig. 1:**
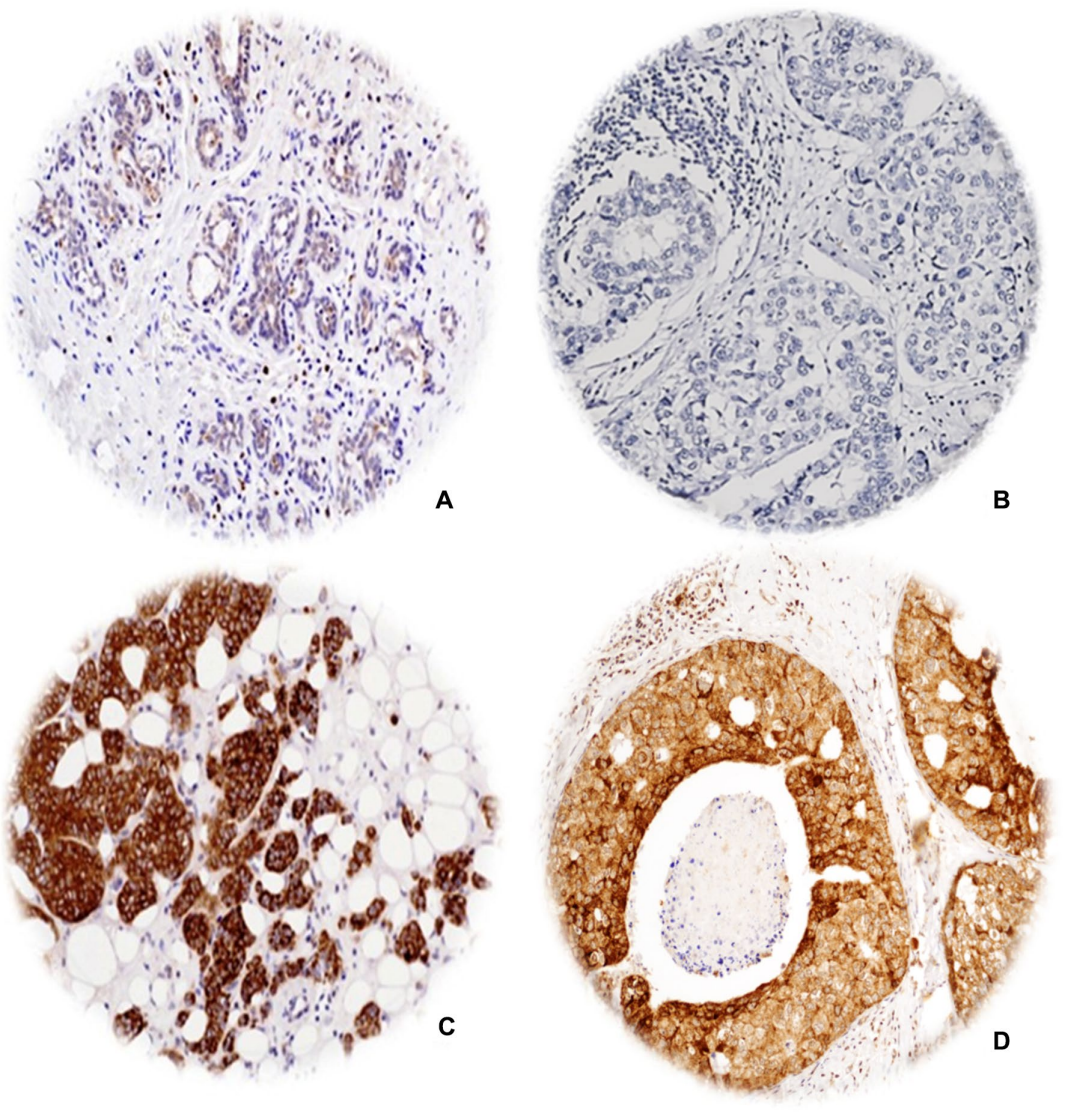
Photomicrographic images (× 40) for immunohistochemical protein expression of IDH2 in breast tissue microarray images; **a** normal breast terminal duct-lobular showing negative to faint staining, **b** negative expression in invasive breast carcinoma, **c** positive expression in invasive breast carcinoma, and **d** positive expression in DCIS

The full face tissue sections demonstrated an even staining of IDH2 expression within IBC and DCIS, indicating that TMA cores are representative for the whole tumour when assessing IDH2 expression. Adjacent normal breast terminal duct-lobular units showed occasional weak cytoplasmic staining. Occasional stained inflammatory cells and stromal fibroblasts were evident in a few cores. When present, IDH2 was expressed in the cytoplasm of epithelial tumour cells (Fig. 1). After exclusion of uninformative cores, 512 and 859 cases were available to evaluate the immunoreactivity in DCIS and IBC, respectively. There was a strong concordance between both observers in IDH2 immunoscoring (ICC = 0.855, *p* < 0.001). Distribution of IDH2 expression showed unimodal distribution. High IDH2 expression was observed in 49% and 59% of pure DCIS and IBC cases, respectively. IDH2 expression was higher in IBC than DCIS (Z = 9.5, *p *< 0.0001).

### Significance of IDH2 protein expression

**Fig. 2:**
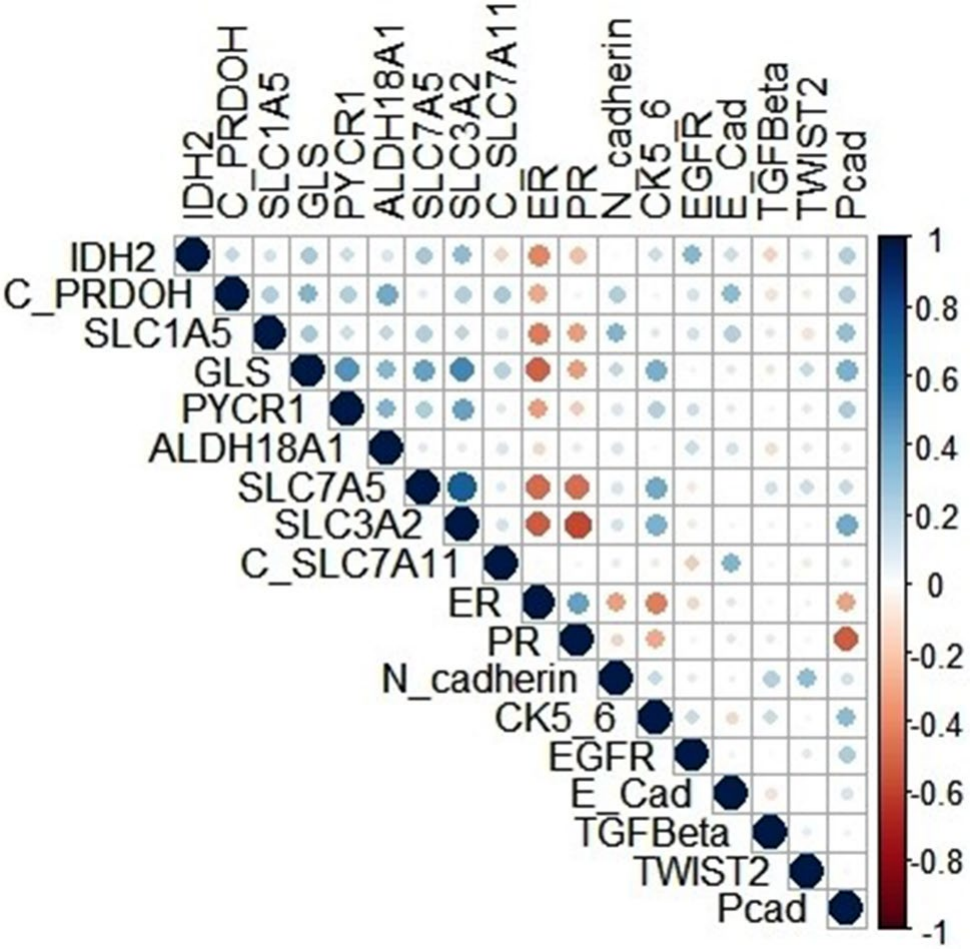
Correlation matrix showing the association between IDH2 protein expression and with other biomarkers related to cellular proliferation, metabolism and epithelial mesenchymal transition. Positive correlation is displayed in blue colour and negative correlation in red colour. Colour intensity is proportional to the correlation coefficient

In pure DCIS, numerous clinicopathological parameters indicating poor DCIS prognosis were associated with high IDH2 expression (Table 1) including younger age at diagnosis, (*p *= 0.035), larger tumour size, higher nuclear grade (both *p *< 0.0001), presence of comedo type necrosis (*p *= 0.001), hormonal receptor negativity and HER2 positivity (all *p *< 0.0001). In IBC, high IDH2 protein expression was associated with positive LVI status (*p *= 0.002), high histological grade, high Nottingham Prognostic Index (NPI), hormonal receptor negativity, HER2 positivity (all *p *< 0.0001), large tumour size (*p *= 0.005), basal phenotype (*p *= 0.007) and high proliferation index (Ki67) (*p *= 0.008) (Table 2). There was positive correlation between IDH2 protein expression and the expression of EGFR (*p *< 0.0001), CK5 (*p *= 0.003), EMT markers including E-cadherin (*p *= 0.003), N-cadherin, P-cadherin and TWIST2 (all *p *< 0.0001). There was a positive correlation between IDH2 and enzymes within the glutamine-proline regulatory axis: GLS (*p *< 0.001), PRODH (*p *= 0.010), ALDH4A1 (*p *= 0.012), and solute transporters including SLC1A5 (*p *= 0.002), SLC3A2 and SLC7A5 (both; *p *< 0.001). Correlation matrix of IDH2 with other associated proteins in IBC is shown in Fig. 2.

**Table 1:** Statistical associations between IDH2 protein expression with clinicopathological parameters in the pure ductal carcinoma in situ cohort

Clinicopathological parameters	IDH2 expression		*χ*^2^ (*p* value)
	Low (*N* = 261) *N* (%)	High (*N* = 251) *N* (%)	
Age (years)			4.4 **(0.035)**
≤ 50	22 (8)	36 (14)	
> 50	239 (90)	215 (86)	
DCIS size (mm)			22.5 **(< 0.0001)**
≤ 20	157 (61)	101 (40)	
> 20	99 (39)	150 (60)	
Nuclear grade			18.8 **(< 0.0001)**
Low	49 (19)	20 (8)	
Moderate	74 (28)	56 (22)	
High	138 (53)	175 (70)	
Comedo necrosis			10.5 **(0.001)**
No	114 (44)	75 (30)	
Yes	147 (56)	176 (70)	
Oestrogen receptor			12.0 **(0.001)**
Negative	36 (18)	74 (33)	
Positive	160 (82)	148 (67)	
Progesterone receptor			18.0 **(< 0.0001)**
Negative	54 (30)	115 (50)	
Positive	129 (70)	114 (50)	
HER2 status			12.7 **(< 0.0001)**
Negative	172 (86)	157 (72)	
Positive	28 (14)	62 (28)	
Molecular classes			20.1 **(< 0.0001)**
Luminal A	89 (63)	75 (39)	
Luminal B	23 (16)	47 (24)	
HER2 enriched	9 (6)	29 (15)	
Triple negative	20 (15)	42 (22)	

Significant *p* values are in bold

**Table 2:** Statistical associations between IDH2 protein expression and the clinicopathological factors in invasive breast cancer cohort

Clinicopathological parameters	IDH2 expression		*χ*^2^ (*p* value)
	Low (*N* = 349) *N* (%)	High (*N* = 510) *N* (%)	
Age (years)			3.526 (0.060)
≤ 50	113 (37)	197 (63)	
> 50	234 (43)	310 (57)	
Tumour size (cm)			7.787 **(0.005)**
≤ 2	199 (45)	241 (55)	
> 2	148 (36)	256 (64)	
Tumour grade			41.664 **(< 0.0001)**
Low	71 (55)	59 (45)	
Moderate	140 (51)	135 (49)	
High	138 (30)	314 (70)	
Tumour stage			1.723 (0.632)
Low	224 (41)	316 (59)	
Moderate	98 (40)	150 (60)	
High	27 (39)	42 (61)	
Nottingham Prognostic Index			21.763 **(< 0.0001)**
Poor	45 (33)	93 (67)	
Moderate	169 (37)	293 (63)	
Good	133 (53)	120 (47)	
LVI status			9.552 **(0.002)**
Negative	250 (45)	311 (55)	
Positive	97 (34)	192 (66)	
Nodal status			0.262 (0.609)
Negative	222 (41)	315 (59)	
Positive	125 (40)	191 (60)	
Oestrogen receptor			25.640 **(< 0.0001)**
Negative	55 (26)	158 (74)	
Positive	292 (46)	350 (54)	
Progesterone receptor			18.100 **(< 0.0001)**
Negative	111 (32)	236 (68)	
Positive	229 (47)	262 (53)	
HER2 status			9.627 **(0.002)**
Negative	312 (43)	415 (57)	
Positive	28 (27)	76 (73)	
Molecular classes			33.632 **(< 0.0001)**
Luminal A	109 (53)	95 (47)	
Luminal B	122 (44)	158 (56)	
HER2 enriched	28 (27)	76 (73)	
Triple negative	43 (28)	112 (72)	
Basal phenotypes			
Negative	272 (43)	366 (57)	7.360 **(0.007)**
Positive	63 (32)	135 (68)	
Ki67			
Negative	127 (47)	142 (53)	7.131 **(0.008)**
Positive	156 (37)	266 (63)	

Significant *p* values are in bold

### IDH2 protein expression and patient outcome

**Fig. 3:**
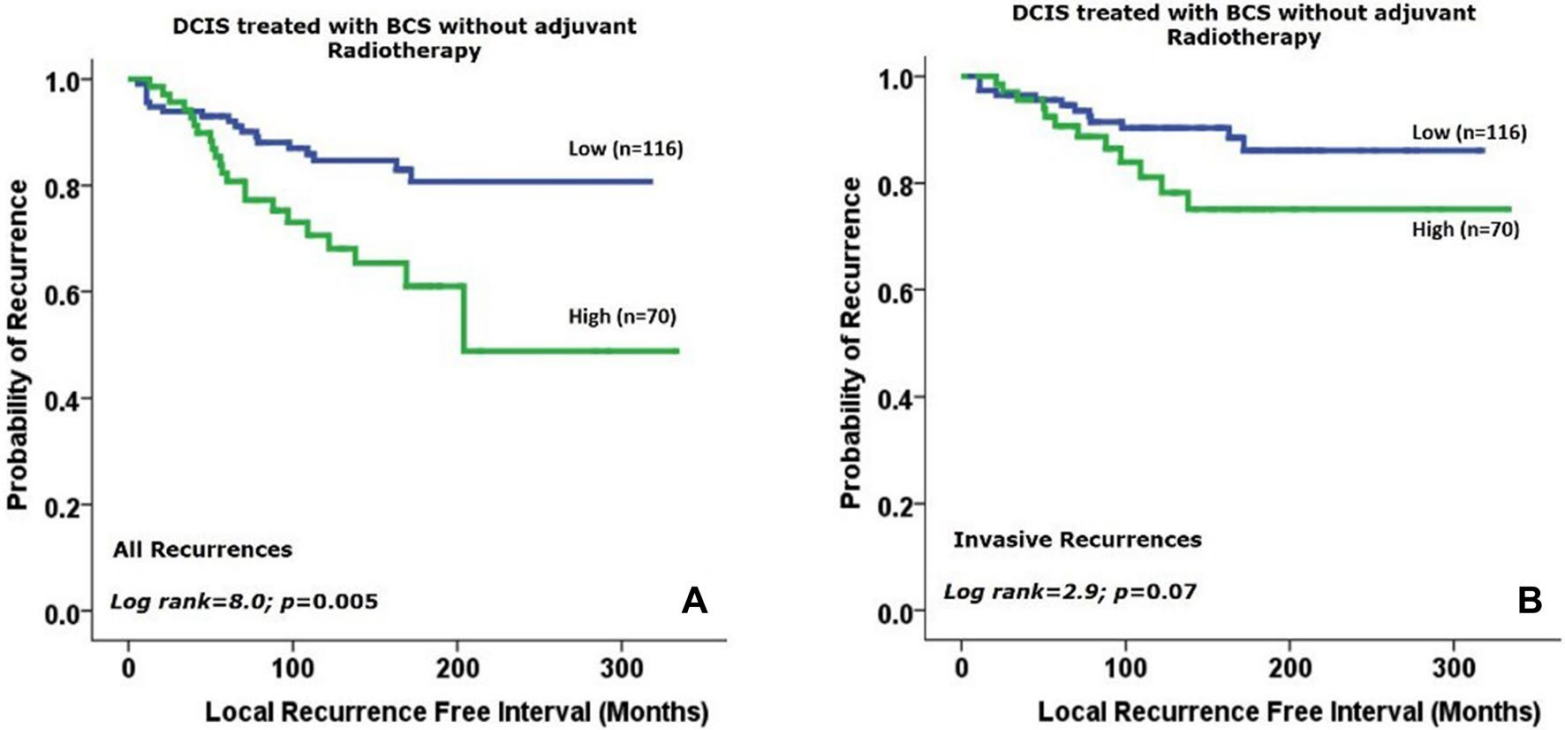
Kaplan–Meier survival plots showing that higher expression of IDH2 is associated with shorter local recurrence free interval in DCIS patients treated with breast conserving surgery for all recurrences (**a**) and a trend with invasive recurrences (**b**)

Survival analysis in DCIS revealed that higher IDH2 expression was correlated with shorter LRFI for all recurrences (HR = 2.4, 95% CI 1.3–4.5, *p *= 0.005, Fig. 3a) and a trend for association with shorter LRFI for invasive recurrences (HR = 1.9, 95% CI 0.9–4.4, *p *= 0.07 Fig. 3b) in patients treated solely with BCS without adjuvant radiotherapy. Multivariate survival analysis revealed that a high expression of IDH2 was an independent poor prognostic factor for DCIS recurrence (HR 2.0; 95% CI 1.1–3.9; *p *= 0.034) regardless of the other variables including patient age at diagnosis, DCIS size, nuclear grade, presence of comedo necrosis and surgical margin width (Table 3).

**Table 3:** Multivariate Cox regression survival analysis of variables (with and without IDH2) predicting outcome in terms of ipsilateral local recurrences in all patients treated by breast conserving surgery in pure ductal carcinoma in situ cohort

Parameters	Hazard ratio (HR)	95% confidence interval (CI)		*p* value
		Lower	Upper	
Conventional clinicopathological parameters associated with high-risk DCIS				
Patient age	0.3	0.1	0.5	**0.0001**
DCIS size	1.6	0.9	2.7	0.055
DCIS nuclear grade	1.8	1.2	2.7	**0.002**
Comedo necrosis	0.6	0.4	1.1	0.096
Margin status	0.8	0.7	0.9	**0.012**
Expression of IDH2 and other clinicopathological parameters associated with high-risk DCIS				
IDH2 expression	2.0	1.1	3.9	**0.034**
Patient age	0.2	0.1	0.4	**< 0.0001**
DCIS size	1.6	0.8	3.3	0.224
DCIS nuclear grade	1.3	0.8	2.4	0.315
Comedo necrosis	1.5	0.7	3.3	0.345
Margin status	0.8	0.7	0.9	**0.039**

Significant *p* values are in bold

**Fig. 4:**
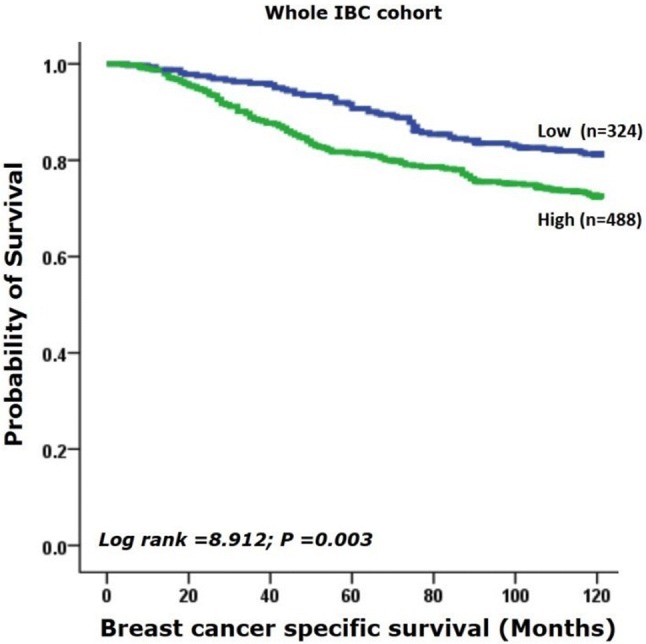
Kaplan–Meier survival plot showing high expression of IDH2 protein is associated with shorter breast cancer-specific survival in the invasive breast cancer cohort

Comparable results were obtained in IBC where high IDH2 protein expression was associated with shorter BCSS *(*HR 1.6; 95% CI 1.2–2.2; *p *= 0.003; Fig. 4). High expression of IDH2 however was not associated with the distant metastasis (HR 1.1; 95% CI 0.9–1.4; *p *= 0.310; Supplementary Fig. 2). When the cohort was split into the intrinsic molecular subtypes, high expression of IDH2 protein in luminal B-like was associated with poor outcome in LVI positive tumours (HR 2.1; 95% CI 1.0–4.5; *p *= 0.044; Supplementary Fig. 3a). HER2 positivity was in borderline association with overexpression of IDH2 in LVI positive status (*p *= 0.062; Supplementary Fig. 3b), but not with TN subtype defined as ER−, PR−, HER2− (*p *= 0.221; Supplementary Fig. 3c).

In multivariate Cox regression analysis, high IDH2 protein expression was an independent predictor of shorter BCSS (HR 1.4; 95% CI 1.1–1.9*; p *= 0.042) regardless of the tumour grade, lymph node stage and nodal status (Table 4).

**Table 4:** Multivariate Cox regression for predictors of breast cancer-specific survival and IDH2 protein expression in invasive breast cancer cohort

Parameter	Hazard Ratio (HR)	95% confidence interval (CI)		*p* value
		Lower	Upper	
IDH2 overexpression	1.4	1.1	1.9	**0.040**
Tumour grade	2.0	1.6	2.7	**< 0.0001**
Tumour stage	2.4	2.0	2.9	**< 0.0001**
Nodal Status	0.4	0.2	0.7	**0.002**

Significant *p* values are in bold

### *IDH2* mRNA expression

**Fig. 5:**
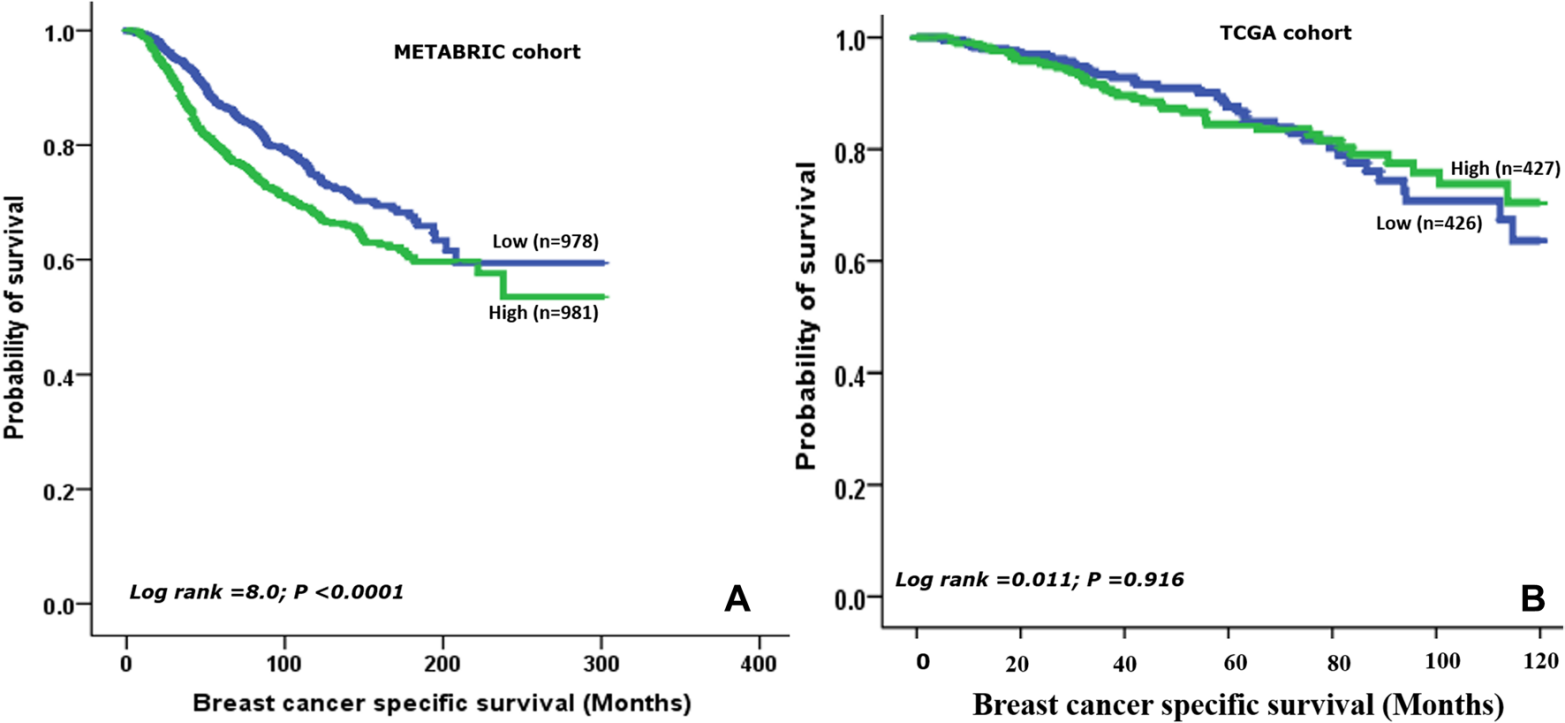
Kaplan–Meier survival plots showing the association between mRNA *IDH2* and breast cancer-specific survival in (**a**) METABRIC cohort and (**b**) TCGA dataset

High *IDH2* mRNA expression was observed in 50% of IBC in the METABRIC and TCGA datasets, respectively. There was a significant positive linear correlation between IDH2 protein expression and *IDH2* mRNA expression (r = 0.240, *p *= 0.002) when tested in the Nottingham subset of the METABRIC cases (*n* = 288). Similar to IDH2 protein, in the METABRIC and TCGA datasets, overexpression of *IDH2* mRNA was significantly associated with LVI-positivity (all; *p *= 0.001). In both datasets, high expression of *IDH2* mRNA was associated with high histological grade and hormonal receptor negativity (both; *p *< 0.0001). Moreover, in the METABRIC cohort, high *IDH2* mRNA levels were associated with large tumour size (*p *= 0.038), axillary lymph node positivity and HER2 positivity (all; *p *< 0.0001); (Table 5). In the METABRIC cohort, BCSS of patients with high *IDH2* mRNA expression was significantly shorter than those with low expression (HR 1.38; 95% CI 1.2–1.6; *p *< 0.0001) (Fig. 5a), but not in TCGA dataset (HR = 1.0; 95% CI 0.7–1.5; *p *= 0.916); (Fig. 5b). External validation using the Gene Miner datasets (*n* = 4039) of IDH2 further supported that high expression of *IDH2* mRNA was positively associated with shorter patient outcome (HR 1.26; 95% CI 1.1–1.4; *p *= 0.0002), Supplementary Fig. 4.

**Table 5:** Statistical association between *IDH2* mRNA expression and clinicopathological parameters in the METABRIC cohort of invasive breast cancer (*n* = 1980) and TCGA Breast Carcinoma cohort (*n* = 854)

Clinicopathological parameters	METABRIC Cohort		*χ*^2^	TCGA Cohort		*χ*^2^ (*p* value)
	Low (*N* = 991) *N* (%)	High (*N* = 989) *N* (%)	(*p* value)	Low (*N* = 427) *N* (%)	High (*N* = 427) *N* (%)	
Age (years)			8.249			
≤ 50	186 (44)	238 (56)	**(0.004)**	322 (52)	301 (48)	2.617 (0.106)
> 50	805 (52)	751 (48)		105 (45)	126 (55)	
Tumour size (cm)			4.300 **(0.038)**			
≤ 2	333 (53)	289 (47)		302 (49)	313 (51)	0.703 (0.402)
> 2	649 (48)	689 (52)		125 (52)	114 (48)	
Tumour grade			151.170 **(< 0.0001)**			
Low	116 (68)	54 (32)		63 (71)	26 (29)	73.783 **(< 0.0001)**
Moderate	484 (63)	286 (37)		228 (61)	147 (39)	
High	340 (36)	612 (64)		116 (33)	236 (67)	
LVI status						
Negative	505 (54)	425 (46)	11.663 **(0.001)**	303 (54)	256 (46)	11.440 **(0.001)**
Positive	289 (45)	346 (55)		124 (42)	171 (58)	
Nodal status						
Negative	557 (54)	478 (46)	12.852 **(< 0.0001)**	214 (50)	212 (50)	0.029 (0.864)
Positive	429 (46)	509 (54)		210 (50)	213 (50)	
Oestrogen receptor						
Negative	109 (23)	365 (77)	182.459 **(< 0.0001)**	51 (28)	134 (72)	45.967 **(< 0.0001)**
Positive	882 (59)	624 (41)		357 (56)	282 (44)	
Progesterone receptor		583 (62)	104.319 **(< 0.0001)**	89 (33)	183 (67)	45.957 **(< 0.0001)**
Negative	357 (38)			316 (58)		
Positive	634 (61)	406 (39)			230 (42)	
HER2 status			123.275 **(< 0.0001)**			
Negative	949 (55)	784 (45)		305 (54)	262 (46)	19.848 **(< 0.0001)**
Positive	42 (17)	205 (83)		43 (32)	0 (68)	
Molecular classes			279.615 **(< 0.0001)**	Not available		
Luminal A	457 (64)	261 (36)				
Luminal B	287 (59)	201 (41)				
HER2 enriched	30 (12.5)	210 (88)				
Basal like	92 (28)	237 (72)				
Normal like	124 (62)	75 (38)				

Significant *p* values are in bold

## Discussion

Breast cancer represents a group of heterogeneous diseases that vary in their morphological, molecular, and clinical behaviour. This heterogeneity poses challenges in precise understanding of the biology of BC, and hence to define a personalised therapy approach [31].

Despite the breakthrough of the genetic and molecular analysis, the mechanisms underlying progression of breast in situ lesions into invasive disease, and those involved in distant metastasis and LVI are still to be defined. Wild-type *IDH2* was previously described as a key factor in DCIS progression into invasive disease [9, 10], and controlling LVI [8]. However, the prognostic significance of IDH2 in breast cancer has not been described before. To the best of our knowledge, this is the first study addressing the role of wild-type IDH2 protein in BC using IHC and well annotated cohort of patients.

The current study included large cohorts of pre-invasive and IBC to assess the transcriptomic and proteomic expression of IDH2 expression and its correlation with the clinicopathological parameters and patients’ outcome. Our analysis of IDH2 expression in DCIS supported our hypothesis that this protein would be associated with features of high-risk DCIS. In addition, the poor prognostic significance of higher IDH2 expression was independent from other clinical and morphological features, and with a trend of association towards invasive recurrence and progression.

Similarly, results in IBC showed that a high IDH2 expression was associated with criteria of aggressive behaviour including LVI, larger tumour size, higher grade and poor NPI. This supports the results of previous studies which demonstrated that IDH2 is significantly associated with LVI [32]. High IDH2 expression was also an independent predictor of shorter BCSS either in proteomic or transcriptomic datasets. In METABRIC and bc-Exminer, datasets support the poor prognostic significance of high IDH2 expression.

Cellular energy produced by the TCA cycle is upregulated in highly proliferative and metastasised cancer cells. Energy metabolism and biosynthetic intermediates such as Alpha-ketoglutarate (αKG) produced through TCA from isocitrate by IDH2 is essential in tumour progression and metastasis. Wild-type IDH2 can reduce αKG and increase 2-HG production, in turn disrupting normal epigenetic regulation of transcription [15]. For example, in hypoxic condition in BC, IDH2 carboxylates α-KG from glutamine to citrate and elevates 2-HG levels, acting as an oncometabolite [33]. Moreover, when IDH2 reduces αKG, it can have an oncogenic impact on cellular differentiation [17]. αKG may have a role in the epithelial–mesenchymal transition (EMT), which is a key step during metastasis. It has been shown that blocking of αKG inhibits cellular transformation and cancerous cell invasion through transamination or reverse TCA cycle [12, 34]. Our data shows that IDH2 is associated with EMT proteins and factors including in cellular metabolism and energy production, which support its role in disease progression and metastasis. This study showed that high IDH2 protein was significantly associated with EGFR which can regulate epithelial mesenchymal transition (EMT), migration and invasion [35]. Moreover, this study also demonstrates that high N-cadherin is associated with high IDH2 protein. Previous studies elucidate that N-cadherin has a role in motility, invasion and metastasis by increasing MMP-9 production allowing tumour cells to penetrate the matrix barriers and to adhere to the endothelium. This may also increase the chance of tumour cells to enter and exit the vasculature [36]. Additionally, it has been reported that N-cadherin can induce EMT which plays a fundamental role in the invasion and metastasis of cancer cells [37]. Thus, the observed association between the high levels of IDH2 and N-cadherin explain the role of IDH2 in LVI as N-cadherin can promote two properties of the metastasised cell i.e. adhesion and invasion. IDH2 was also associated with TWIST2 EMT marker. EMT has a highly important role in cancer invasion and metastasis. In BC and lymph node metastases, Twist2 is overexpressed as it results in morphological transformation, downregulation of epithelial markers and upregulation of mesenchymal markers [38]. Thus, Interaction with EMT markers explains the role of IDH2 in DCIS progression into invasive disease and invasion of BC cells through the lymphovascular channels. EMT is a known mechanism attained by the breast carcinoma cells for basement membrane invasion and LVI [39]. Further mechanistic studies are highly warranted to understand the underlying molecular mechanisms.

Additionally, one of the major hallmarks of cancer is reprogramming energy metabolism [40]. High rates of glucose and glutamine are taken up by cancer cells to produce NADPH to survive and grow followed by decreasing the level of intracellular αKG [41]. Many studies provide evidence that oncogenes can alter cancer cell metabolism including glutamine transporters which were associated with high IDH2 in this study [26]. This may imply that IDH2 could potentially re-programme the metabolic pathways in BC and support tumour cells to survive and indicate its prognostic significance in BC. Our observations showed that high IDH2 was associated with high grade DCIS and IBC with a highly proliferative index. High expression of IDH2 was associated with EGFR, CK5 and CK14 which are connected to the highly proliferative basal phenotype [42, 43]. In this study, IDH2 was also associated with high Ki67 expression reflecting increased cellular proliferation [44]. It has been reported that IDH2 has a critical role in cell proliferation through the alteration of NADP levels. Beside the role of NADP as antioxidants, it has been reported that NADP has an important role in cell death. It links cell survival with biological properties such as energy metabolism and oxidative stress, which are factors that determine cell death [14]. Primary tumour cells must proliferate and invade the adjacent tissue to establish an invasion and metastasis cascades, which consists of many steps including basement membrane degradation and LVI. Proliferation lasts until the invasion of blood vessels or lymphatic channels occur. Tumour cells at this stage evade apoptosis and immune responses [45]. Thus, IDH2 may have an important role in cell proliferation, which is a perquisite step of the invasion and metastatic process, which can lead to the development of invasive cancer from a pre-invasive lesion, and for development of LVI and hence distant metastasis.

Furthermore, *IDH2* mRNA and protein were also highly expressed in TNBC and HER2+ either in IBC or DCIS, in concordance with previous studies [5]. HER2+ tumours were more likely to have IDH2 protein which is perhaps unsurprising as high grade progressing DCIS and LVI are significantly associated with HER2 positivity [1]. It also has been shown that tumour microenvironment plays a major role in the HER2 signalling pathway, invasion and the development of LVI, which is a crucial step in metastasis [46]. IDH2 high expression might enhance HER2 signalling pathways that can have effect on the tumour microenvironment to support the growth of the tumour cell, stimulating invasion, LVI and metastasis in BC. The molecular subtypes differ from each other in their expression of claudin [47]. For example, low expression of the claudin tight junction protein and the high expression of proteins involved in EMT and cancer cell invasiveness were reported in TN compared to other subtypes [48]. This could explain the difference in the significance between these subtypes.

This study reveals that IDH2 is associated with poor prognostic characteristics and short-term survival outcomes in BC including higher local recurrence rate after diagnosis of DCIS or poor survival rate in IBC. Furthermore, a positive association of IDH2 and elevated levels of basal cytokeratin confers a poor prognosis. Basal cytokeratins are strongly associated with high histological grade, negative hormone status and worse patient outcome [39, 49]. Among subgroups, overexpression of IDH2 protein appears to play a particularly significant role in luminal B subtype which is in concordance with a recent study [50].

In conclusion high expression of IDH2 is an independent poor prognostic factor in BC. High expression of IDH2 may have a key role in BC progression from DCIS to IBC and in the development of LVI and metastasis. Further functional assessment of IDH2 in BC is warranted to detect its underlying mechanistic roles and its therapeutic potential.

## Electronic supplementary material

Below is the link to the electronic supplementary material.

Supplementary material 1 (PDF 457 kb)

## Data Availability

The authors confirm the data that has been used in this work is available on reasonable request.
