# Supplementary material for: The prognostic significance of wild-type isocitrate dehydrogenase 2 (IDH2) in breast cancer
Source: Breast Cancer Res Treat. 2019 Oct 10;179(1):79–90. doi: 10.1007/s10549-019-05459-7 (PMC6985218; doi:10.1007/s10549-019-05459-7)
Supplement: Supplementary file 1 — Supplementary material 1 (PDF 457 kb) [file 10549_2019_5459_MOESM1_ESM.pdf]

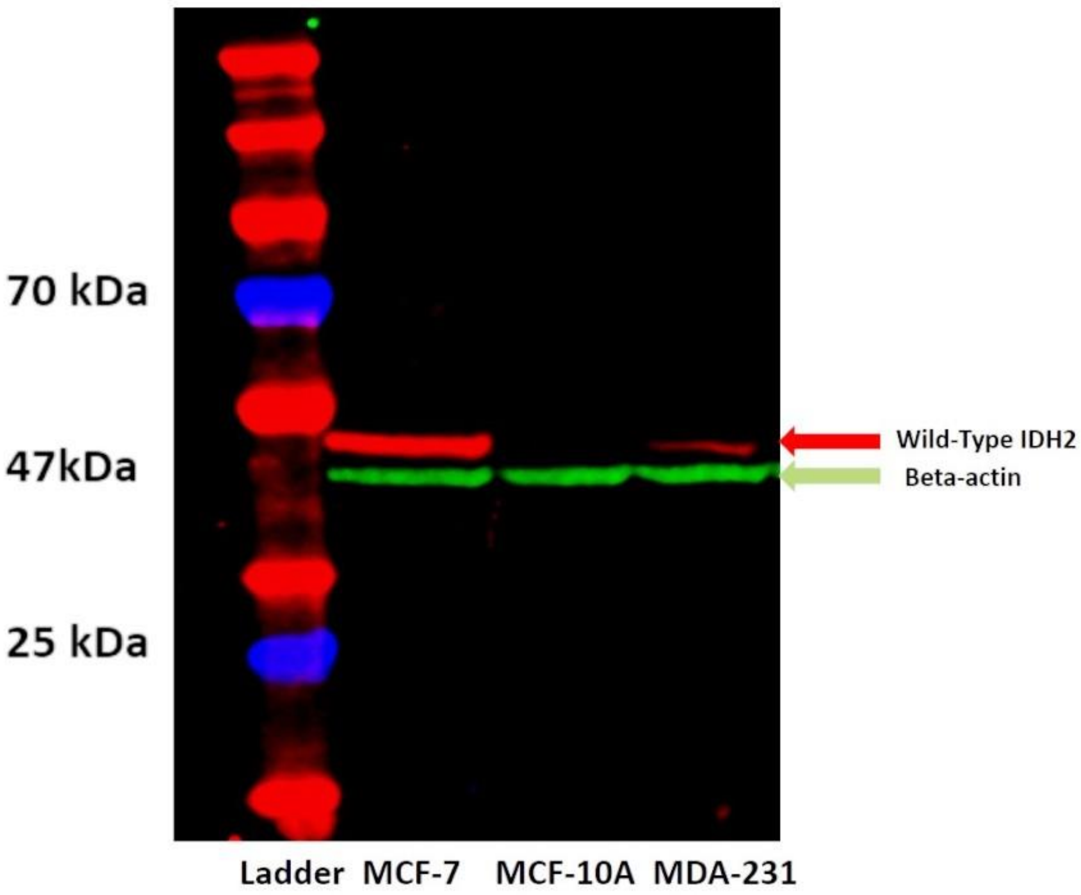

Supplementary Figure (1): Western blotting image showing a single specific band for IDH2 protein antibody at the predicted molecular weight (~47KDa).

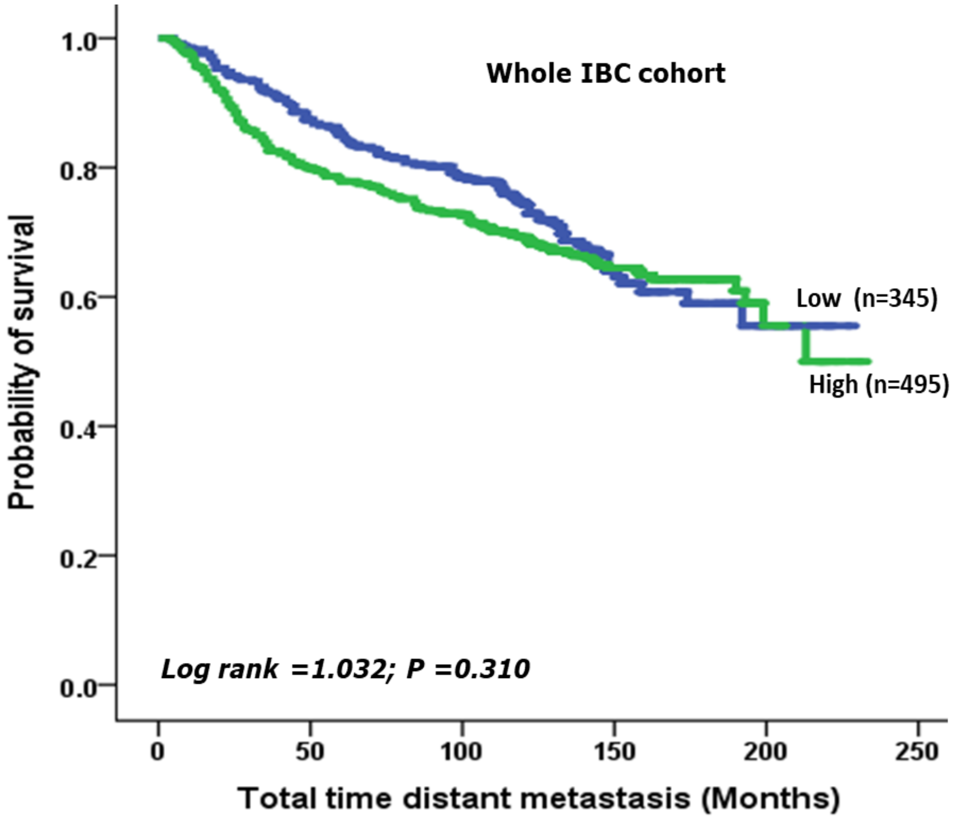

Supplementary Figure (2): Kaplan–Meier survival plot showing the association between the IDH2 protein expression and distance metastasis free survival

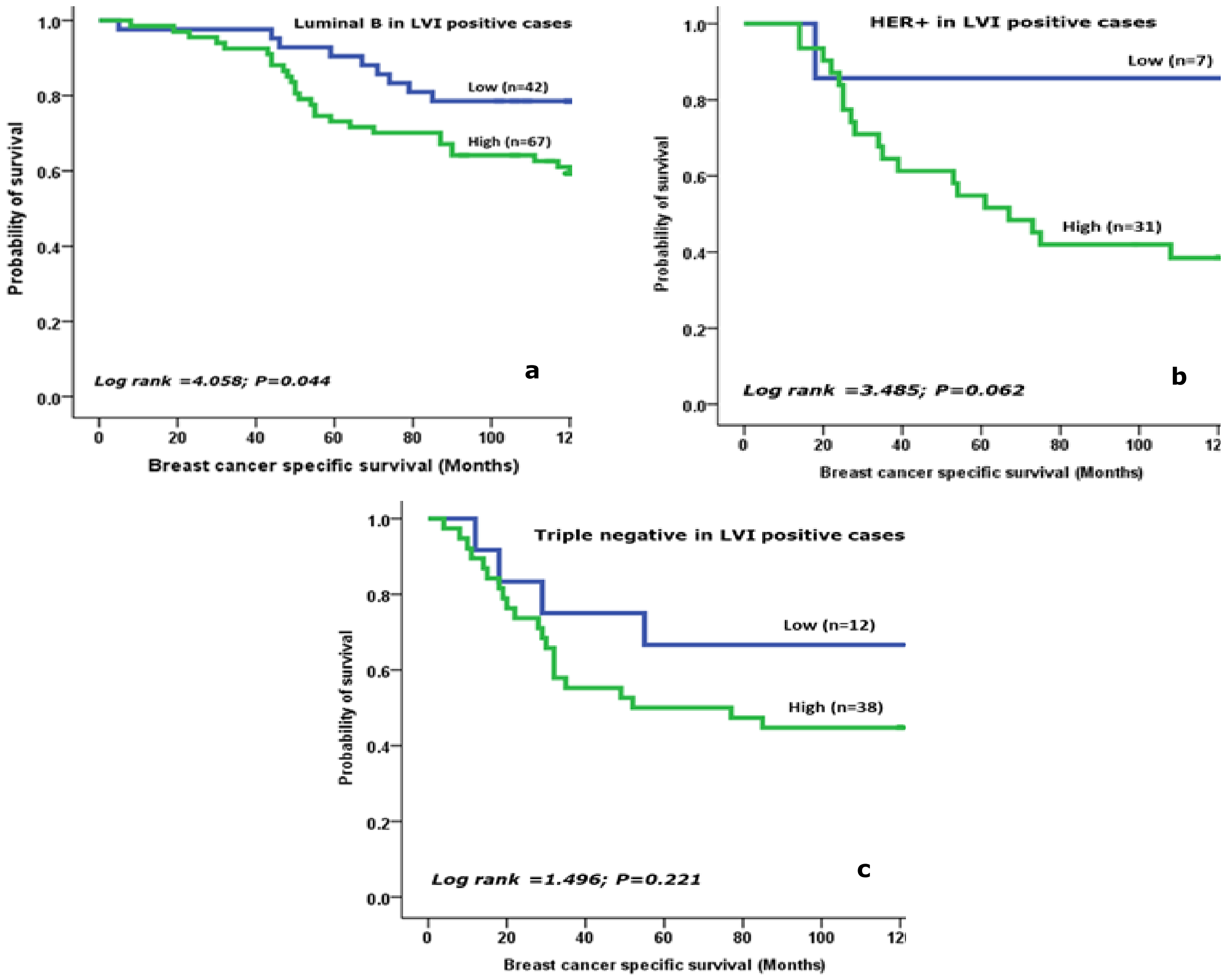

Supplementary Figure (3): Kaplan–Meier survival plots showing the association between of IDH2 protein in different molecular classes in invasive breast cancer with positive lymphovascular invasion and the breast cancer specific survival in; a) luminal B b) HER2 enriched, and c) triple negative breast cancer groups.

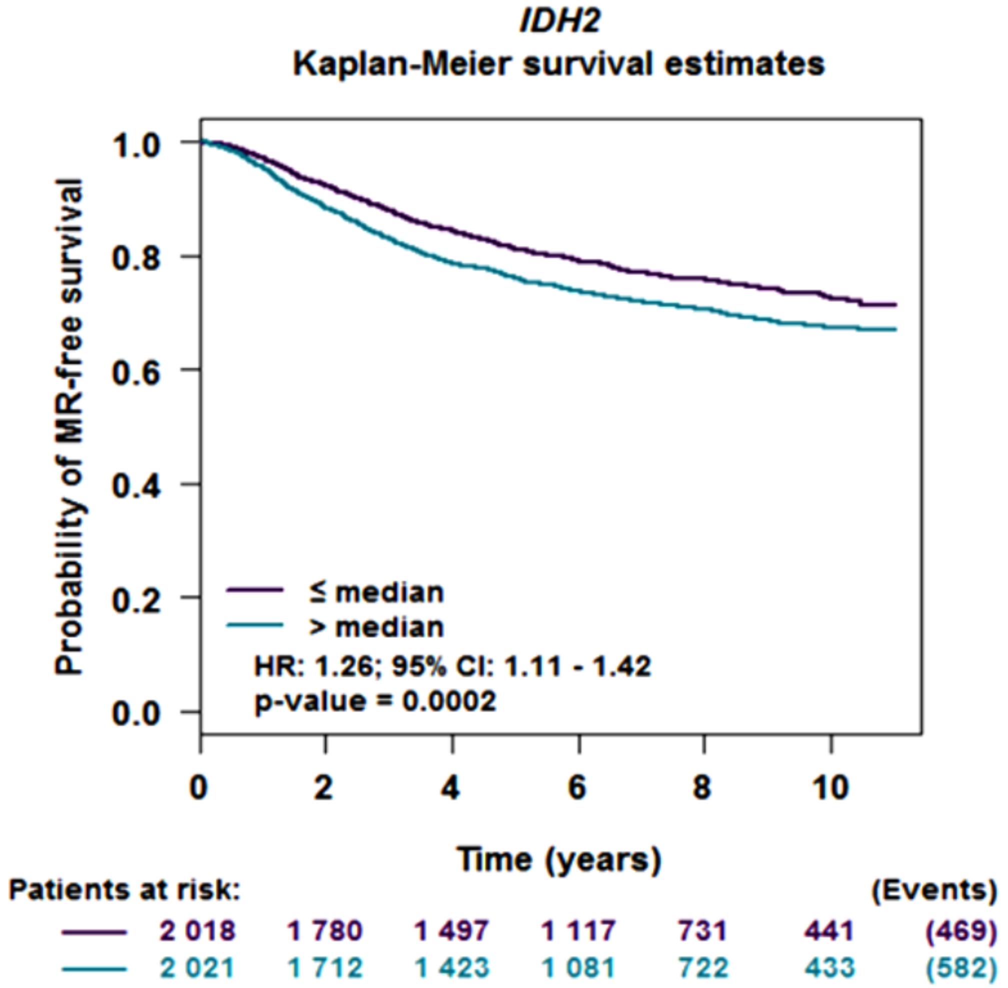

Supplementary Figure (4): Kaplan–Meier survival plots showing the association between *IDH2* mRNA and patients' outcome in publicly available dataset; the (n=4039) Breast Cancer Gene-Expression Miner v4.2 (bc-GenExMiner v4.2)
